# Quantitative genetic analysis deciphers the impact of cis and trans regulation on cell-to-cell variability in protein expression levels

**DOI:** 10.1371/journal.pgen.1008686

**Published:** 2020-03-13

**Authors:** Michael D. Morgan, Etienne Patin, Bernd Jagla, Milena Hasan, Lluís Quintana-Murci, John C. Marioni

**Affiliations:** 1 Wellcome Sanger Institute, Wellcome Genome Campus, Hinxton, Cambridge, United Kingdom; 2 Cancer Research UK–Cambridge Institute, Robinson Way, Cambridge, United Kingdom; 3 Human Evolutionary Genetics Unit, Institut Pasteur, CNRS UMR2000, Paris, France; 4 Cytometry and Biomarkers UTechS, Institut Pasteur, Paris, France; 5 Hub Bioinformatique et Biostatisque, Départment de Biologie Computationalle—USR 3756 CNRS, Institut Pasteur, Paris, France; 6 Human Genomics and Evolution, Collège de France, Paris, France; 7 EMBL-EBI, Wellcome Genome Campus, Hinxton, Cambridge, United Kingdom; Stanford University School of Medicine, UNITED STATES

## Abstract

Identifying the factors that shape protein expression variability in complex multi-cellular organisms has primarily focused on promoter architecture and regulation of single-cell expression *in cis*. However, this targeted approach has to date been unable to identify major regulators of cell-to-cell gene expression variability in humans. To address this, we have combined single-cell protein expression measurements in the human immune system using flow cytometry with a quantitative genetics analysis. For the majority of proteins whose variability in expression has a heritable component, we find that genetic variants act *in trans*, with notably fewer variants acting in *cis*. Furthermore, we highlight using Mendelian Randomization that these variability-Quantitative Trait Loci might be driven by the *cis* regulation of upstream genes. This indicates that natural selection may balance the impact of gene regulation *in cis* with downstream impacts on expression variability *in trans*.

## Author summary

Genetic variation can change how much a gene is turned on or off in a tissue or a population of cells of the same type. However, this averaging of expression levels across a cell population masks an important aspect of gene expression regulation, namely its variability. Recent work in humans has indicated that nearby (*cis*) genetic factors minimally influence this variability. We have combined genetic measurements with flow cytometry single-cell protein levels to resolve the genetic control of gene expression variability in human immune cells. Importantly, we have demonstrated that whilst genetic variants near the target genes (*cis*) rarely influence variability, there is still an extensive genetic contribution from genetic loci faraway, or on a separate chromosome (*trans*). Furthermore, we have resolved that these *trans* genetic effects regulate the expression of other nearby genes, which leads to changes in gene expression variability of our target proteins. Our findings can be explained by an evolutionary balance between the *cis* regulation of gene expression levels, and the downstream consequences on gene expression variability.

## Introduction

Cell-to-cell variability in gene expression levels is a ubiquitous feature of life on earth. This heterogeneity, broadly referred to as expression noise, is a function of transcriptional and translational regulation [1], as well as cellular state and environment [2–5]. The delineation of expression noise into “intrinsic” and “extrinsic” components [6] is mirrored by the separation of genetic influences on gene expression into *cis* and *trans* components. Intrinsic noise represents the differences in promoter output between two alleles of the same gene, whilst extrinsic noise represents all other sources of variability [6]. Intrinsic noise is largely attributed to the stochastic activation of a promoter that produces bursts of mRNA molecules [7]. The consequences of cell-to-cell expression variability (i.e. the sum of all noise sources [8]) manifest as therapeutic resistance in cancer [9,10], environmental adaptation in yeast [11] and prokaryotes [11,12], as well as lineage plasticity in murine T cells [5,13], to highlight just a few examples.

To understand the broader determinants of gene expression variability within and between cells, or organisms, previous studies have used targeted approaches to perturb individual genes [14], or probed how cis regulatory elements influence transcriptional dynamics [15–17], and how this is shaped by sequence variation, notably in yeast [11,14,15,18]. Additional mechanistic studies have uncovered the role of promoter architecture and distal regulatory elements in determining the magnitude of gene expression variability in mammals [19,20]. Moreover, several biological processes have been identified that influence gene expression variability in both prokaryotes and eukaryotes, including nuclear transport and post-transcriptional regulation [1,21]. However, with the exception of a recent CRISPR/Cas9-based screen [22], it has been hard to systematically evaluate the contributions of different biological processes to gene expression variability.

Quantitative genetics, and by extension genome-wide association studies, have been highly successful at providing novel insights into the biological pathways that influence complex phenotypes, including human diseases [23,24], and how they have been shaped by natural selection. We have combined a genome-wide quantitative genetics approach with single-cell protein measurements in the human immune system to elucidate the genetic architecture and regulation of cell-to-cell gene expression variability. Firstly, we demonstrate that expression variability differences between individuals are heritable. Conducting scans for common genetic variation in two independent cohorts of related (TwinsUK) and unrelated individuals (Milieu Intérieur), we identify *trans* genetic influences, distributed across the genome, on 155 protein expression variability traits—which we call variability-pQTLs. Curiously, we note fewer *cis* variability-pQTLs compared to mean expression QTL (97 vs 1210). The enrichment of *trans* variability-pQTLs around protein-coding genes indicates that they may act to influence the expression and dynamics of nearby genes in *cis*. Employing a Mendelian Randomization (MR) analysis we highlight specific examples where *cis*-eQTLs in immune cells contribute to cell-to-cell expression variability. These findings demonstrate the marked skew in *cis* vs. *trans* regulation of cell-to-cell gene expression variability, and suggest an evolutionary trade-off between noise control and the evolution of mean expression levels.

## Results

### A systematic evaluation of protein expression variability across the human immune system

**Fig 1:**
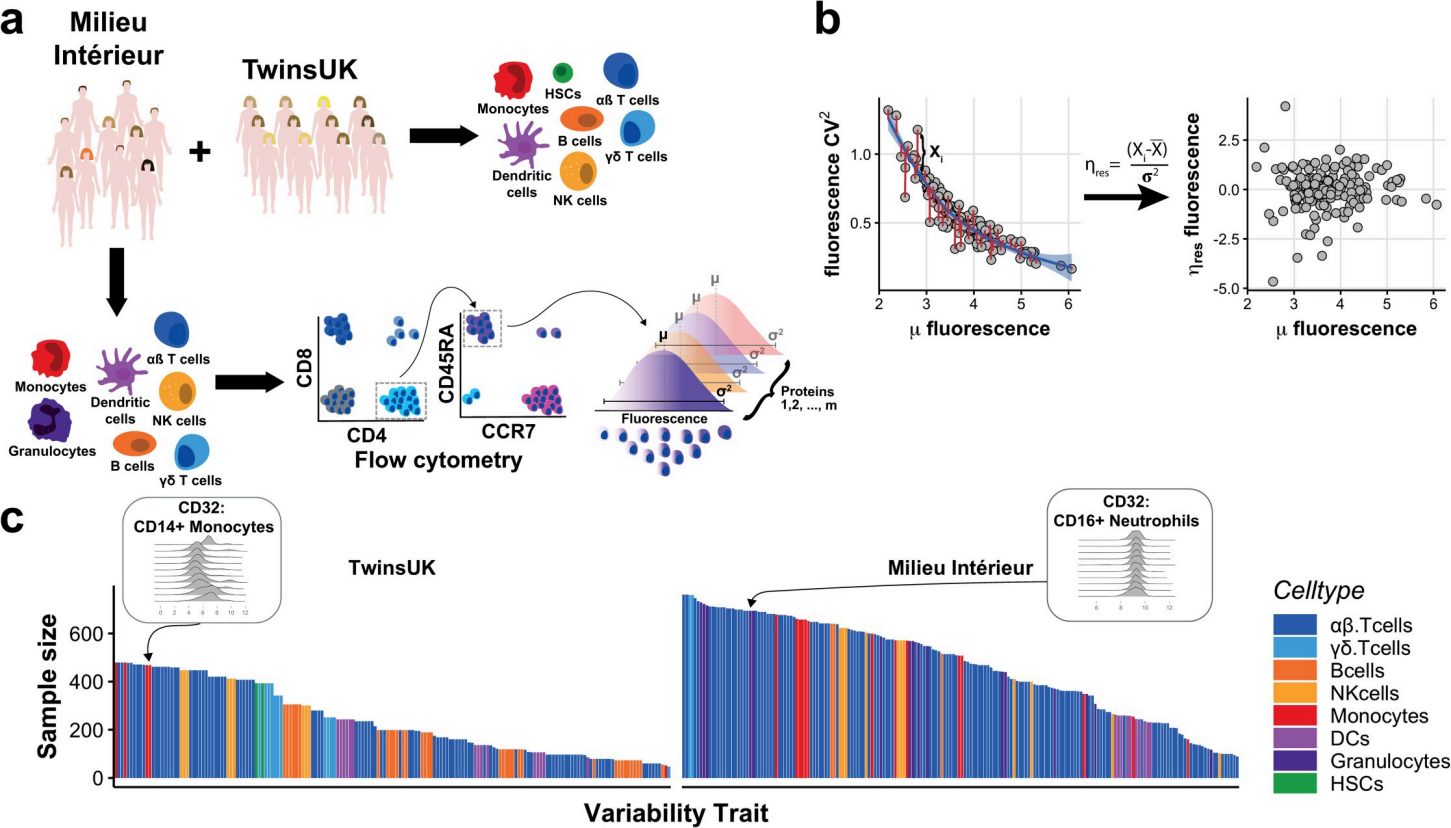
Surveying cell-to-cell protein expression variability across the human peripheral blood immune system. (a) Overview of the experimental design, showing how flow cytometry is used to profile immune cell type populations across multiple individuals from two cohorts. (b) Average expression and cell-to-cell protein expression variability are calculated for each cell type and protein combination (trait) in each individual (grey circles). Variability is quantified by the CV^2^ (y-axis left) which is inversely proportional to mean expression (x-axis). Using a local polynomial fit between the CV^2^ and mean (μ) expression, the mean-adjusted variability is taken as the standardised deviance from this fit (y-axis right, η_res_). (c) Bar charts showing the sample size for each trait in the TwinsUK (left, range 48–479) and Milieu Intérieur cohorts (right, range 89–761) after quality control. Colours denote broad cell type categories—for details of specific cell types see S1 Table.

To quantify cell-to-cell protein expression variability we took advantage of two recently published immune-profiling flow cytometry studies in ~480 human twins (TwinsUK) [25] and ~1000 unrelated individuals from France (Milieu Intérieur) [26]. Flow-cytometry evaluates the expression level of target proteins at single-cell resolution using fluorescence-conjugated antibodies. This provides the ability to simultaneously define cell populations, and measure the cell-to-cell variability within each population across a number of target proteins [27], albeit semi-quantitively. We collated the flow-cytometry measurements across all sets of (previously validated) antibody panels in each study [26], which collectively targeted 47 proteins and 59 different peripheral blood immune cell (sub)types (Fig 1A, S1 Table).

One of the largest known influences on expression variability between single cells is cell volume [28,29]. Therefore, we normalised all single-cell fluorescence measurements by their cell volume, after removing doublets, to remove any individual, technical, environmental or genetic influences on cell size from our study (Methods). Finally, to control for the previously described relationship between variability and gene expression [27] (S1 Note), we used a local polynomial regression to model the relationship between the mean and squared coefficient of variation (CV^2^) across individuals (separately in each cohort). Taking the standardised residuals, η_res_, from this fit, yields a mean-adjusted measure of gene expression variability for each individual that is unconfounded with mean expression (Fig 1B).

Following quality control to remove fluorescence measurements on fewer than 100 cells, (see Methods), we calculated the mean and η_res_ for each individual for whom data were measured for a specific protein:cell-type combination (defined hereafter as a ‘trait’). In total we analysed 171 mean and 171 variability traits in the TwinsUK cohort, and 229 mean and 229 variability traits from the Milieu Intérieur study. This represents the richest survey of cell-to-cell protein expression variability in the human immune system to date (Fig 1C).

### Estimating the influence of genetics and environment on protein expression variability in twins

**Fig 2:**
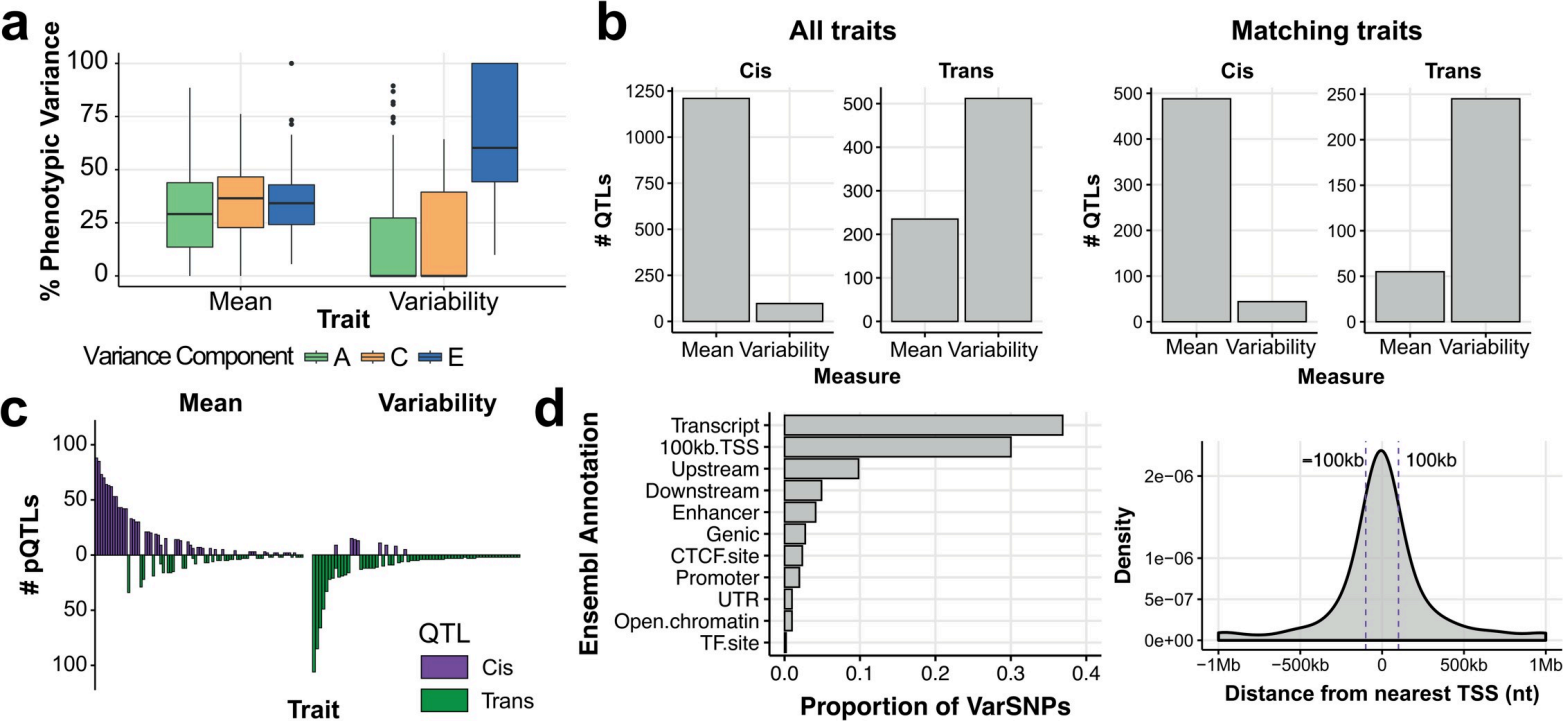
A relative depletion of *cis* genetic control of protein expression variability. (a) Boxplots summarising the variance decomposition of all mean and expression variability traits into additive genetic (A), common environment (C) and unique environmental (E) components. (b) A summary of *cis* and *trans* QTL mapping for mean and variability traits demonstrates a depletion of *cis* variability-pQTLs using all traits tested (left) and the subset of matching traits (right). (c) Bar plots of the numbers of cis and trans QTLs across mean (left) and variability (right) traits illustrates the relative depletion of *cis* regulation of cell-to-cell expression variability. (d) varSNP annotations demonstrate (left) gene-centric association signals and (right) the proximity of varSNPs to the nearest protein-coding gene TSS.

Previous studies have observed inter-individual and inter-strain differences in gene expression variability in yeast and plants [30–32], and identified specific genetic variants that are correlated with protein expression variability in T cells [33]. However, none of these studies quantified the total genetic contribution to expression variability across proteins. Therefore, to estimate the extent to which heritable factors influence protein expression variability, we performed variance components analysis. Leveraging the known genetic relationships between mono and di-zygotic twins in the TwinsUK cohort we estimated the genetic, as well as shared (within family) and unique environmental components, for each of 171 variability traits. As a comparison we applied the same analysis to mean expression for 171 mean traits (Fig 2A, S1–S7 Figs).

Across the majority of variability traits, the unique environmental component is the prime influence, indicating that cell-to-cell expression variability is a consequence of the individual life histories of study participants, as well as experimental, stochastic and technical influences. In particular, the latter includes the non-specific binding of antibodies selected against the target proteins, reflecting a limitation of using indirect fluorescence measurements. Although the shared environment did not contribute to explaining variability in 53.8% of the traits considered, in the remainder its contribution was relatively substantial (median 40.3% of the trait variance). The shared environmental component includes *in utero* effects, as well as shared up-bringing, social and non-additive genetic effects and chronological age. In particular, age has previously been associated with changes in gene expression variability in a number of different cell types and organisms [34], including näive CD4+ T cells [35] (S3 Note, S8–S10 Figs).

For 59/171 (34.5%) of the variability traits the additive genetic component (σ_g_^2^) was significantly greater than 0 (permutation test p-value≤0.05; S11 and S12 Figs). We observed that the genetic contributions to expression variability differ between cell types for the same protein (S13 Fig). The narrow-sense heritability estimates reveal that genetic factors have a broad range of influence on cell-to-cell gene expression variability (median 43%, range 0.019–89%). In comparison, 88.3% (151/171) of mean expression traits have a detectable heritable influence (Fig 2A), with a median contribution of 32% (range 0.01–88.6%). Overall, we have demonstrated that genetic variation contributes to inter-individual differences in protein expression variability in a cell-type specific manner.

### Variability quantitative trait loci mapping

Given these results, we next sought to identify specific genetic loci that could explain the observed heritability. We scanned, separately in each cohort, for genetic variants that influence mean and expression variability in *cis* and in *trans* using a linear mixed model to account for the genetic relationships between individuals [36,37] (S14–S17 Figs). Collectively we tested 380 mean (MI: 229 traits, TwinsUK: 151 traits) and 288 variability traits (MI: 229 traits, TwinsUK: 59 traits) for both *cis* and *trans* effects across both cohorts. After grouping association signals for each trait based on linkage disequilibrium [38,39] (LD clumping), we noted that the number of significant *cis* effects was ~10-fold higher for mean traits than variability (Fig 2B). This was not driven by the larger number of mean traits tested (n = 380 vs. 288), as this difference in number of *cis*-pQTLs remained when we subset to the same trait for both mean and variability (Fig 2B). In comparison, we identified many more *trans* pQTLs for variability traits than we did for mean traits, which likewise was not due to differences in the number of mean and variability traits that we tested (Fig 2B), nor due to a small number of traits with many QTLs (Fig 2C). This imbalance in the genetic architectures of mean and expression variability suggests that between-individual differences in gene expression variability are primarily influenced by *trans* effects. Moreover, when looking at the small number of traits that were measured in both cohorts, the replication rate was greater than expected by chance (binomial test p-value = 6.5x10^-5^; S18 Fig), giving confidence in the robustness of our findings.

To interpret the variability-pQTLs that act in *trans*, we considered all loci across both cohorts and annotated the lead SNPs with the smallest p-value at each locus (henceforth called varSNPs) based on their overlap with regulatory and genome annotations using the Ensembl database. We observed that 36.9% of varSNPs mapped to transcribed regions, with a further 9.8% and 4.9% in upstream and downstream regions, respectively (Fig 2D). We also note a subtle enrichment of varSNPs located within 100kb of the nearest transcriptional start site (TSS) compared to MAF-matched control SNPs (OR 1.33, p-value 0.048; Fig 2D).

### *Cis* genetic modulation of gene expression potentially drives protein expression variability-pQTLs

Our finding that most variability-pQTLs act in *trans* to the measured protein begs the question: what mechanism leads to these cell-to-cell expression level differences? Genetic control of average gene expression levels in *cis* has been the subject of extensive research, revealing widespread cis-regulation of gene expression levels [40–46]. Given this, and based on enrichment of varSNPs around genic regions, we hypothesised that *cis* genetic modulators of mean expression by variability-pQTLs may mediate cell-to-cell fluctuations in levels of the target proteins. To this end, we searched for variability-pQTLs that overlapped with *cis*-eQTLs in equivalent cell types [47–51] (S2 Table). Across matching cell types, we identified 260 *cis*-eQTLs that could be compared with 94 of our *trans* variability-pQTLs (18.4% of all *trans* variability-pQTLs).

**Fig 3:**
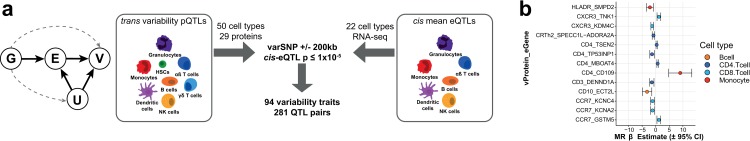
*cis-*eQTLs potentially drive protein expression variability of downstream genes. (a) A schematic of Mendelian Randomisation as a directed acyclic graph (left) and cell type matching between variability pQTLs and mean *cis*-eQTLs (right). G denotes the genetic instrument used to mediate the potential causal relationship between gene expression (E) and protein expression variability (V). Unobserved confounding (U) and the presence of direct or indirect pleiotropy (grey dashed lines) can induce false positive associations. (b) Common genetic predictors between *cis*-eQTLs and variability-pQTLs in human immune cells (shown are those with an FDR < 5%). The x-axis denotes the MR regression estimate (β), error bars denote the 95% CI. Y-axis labels show the vProtein and eGene. Points are coloured by the cell type in which the eGene and variability QTL are both present.

Where concordant SNPs were present in our study and each eQTL study, we used Mendelian Randomisation (MR) analysis between each protein-coding eGene and the protein expression variability trait to infer causality (Fig 3A). Specifically, we tested the hypothesis that the exposure (eGene expression level) is causally associated with the outcome (protein expression variability; vProtein), conditional on the genetic instrument (varSNP) (Fig 3A) using 281 pairs of varSNP and *cis*-eQTL eGenes. Adjusting for multiple testing (FDR 10%), we found that 62.8% (59/94) of tested *trans* variability QTLs could be explained by at least one mean *cis*-eQTL of a different gene (S19 Fig).

These results provide candidate explanatory relationships between *cis*-eQTLs and our *trans* variability-pQTLs. For instance, rs971419521 is associated with increased CD3 variability in CD4+ regulatory T cells (β 1.00, SE 0.17, p = 8.35x10^-9^). We find a common genetic predictor between lower *DENND1A* expression in memory Tregs [47] and increased CD3 variability in Tregs (MR adjusted p-value 2.6x10^-3^, Fig 3B). *DENND1A* encodes DENN/MADD domain containing 1A, a guanosine exchange factor that regulates clathrin-mediated endocytosis [52]. CD3 subunits contain endocytosis signals for internalisation [53–56], which is key for T cell receptor turnover. We therefore speculate that fluctuations in endocytosis may lead to variable levels of CD3 on the surface of regulatory T cells, with the potential to influence regulatory T cell activation.

By integrating *cis*-eQTL information with variability-pQTLs we have highlighted how *cis* gene expression can potentially impact cell-to-cell protein expression variability in *trans*.

## Discussion

Here we have provided insights into the control of cell-to-cell protein expression variability in the human immune system by means of a novel re-analysis of publicly available flow cytometry data. We have presented the first systematic analysis of the impact of genetic factors on cell-to-cell protein expression variability across human cohorts. Notably we have demonstrated that protein expression variability, often referred to as noise, is a heritable and polygenic trait in humans, as it is in yeast [31] and plants [32]. Curiously, the latter reported extensive *trans* variability eQTLs in *Arabidopsis thaliana* for > 20,000 transcripts, but observed that *cis* effects generally had larger effect sizes, more similar to the genetic architecture of mean mRNA levels [57,58]. This contrast with our findings might be explained by genetic regulation of cell type composition within *A*.*thaliana* as has been observed in humans [25,26], or may reflect the larger contribution of *trans* factors to protein levels compared to mRNA [59]. Secondly, our analyses illustrate how cell-to-cell expression variability, for the proteins studied, is primarily shaped by the actions of genetic variants that act *in trans*, suggesting that variability is primarily impacted by the cellular environment, a notion supported by the observation that genetic influences on protein networks are primarily mediated by non-transcriptional mechanisms [59]. Using quantitative genetics and Mendelian Randomisation, we were further able to infer that many of these trans-acting variants, which lie within 100kb of another gene, might function *in cis*. By so doing, they not only influence the expression of the proximal gene, but also impact the wider cellular microenvironment, thereby driving variability of downstream genes. Importantly, whilst we and others have observed a lack of *cis*-genetic effects on variability in humans [60], this does not imply that variability is not regulated *in cis*. Indeed, the study of experimentally induced sequence variation in transcriptional regulatory elements has revealed key mechanisms by which variability is controlled at the molecular level [11,14,15,18]. However, it is crucial to note that whilst common standing genetic variation in humans does not have a large influence on variability in *cis*, at least for the proteins included in this study, this is not the same as saying that there is no influence of *cis*-regulatory elements on variability. Instead, it supports a model whereby any *cis*-regulatory elements that do influence protein expression variability are not altered by common single nucleotide polymorphisms.

Moving forward, we anticipate that one way of increasing power to detect variability-pQTLs will be to obtain a better resolution of cell types both within and across studies. Single-cell RNA-sequencing provides a natural means for doing this, since it is able to profile all expressed genes, providing a more fine-grained ability to cluster cells into physiologically meaningful groups. Moreover, recently developed protocols allow mRNA and cell-surface proteins to be profiled in parallel [61], meaning that variability across multiple regulatory layers can be interrogated. Finally, our study was limited to the 47 proteins included in the original studies; extending these investigations proteome-wide and using larger cohorts will provide a more global picture of the impact of common genetic variants on gene expression variability. Using larger cohorts is especially important since, consistent with Sarkar et al. [60], our power to detect variability pQTLs is highly sensitive to sample size (S4 Note; S37 Fig).

From a broader perspective, our results have implications for our understanding of how natural selection can shape gene expression levels. The lack of genetic variants that act *in cis* to modulate gene expression variability is consistent with the action of purifying selection[14]. However, somewhat counterintuitively, we observe that cis-acting variants can have knock-on effects that manifest themselves *in trans* by increasing variability in expression of downstream genes. Why, if natural selection acts to remove variants that act *in cis*, is this increased variability tolerated? We speculate that there might exist a trade-off between regulating a gene’s expression directly and downstream impacts upon variability of other genes. This complex interplay might explain why variability-eQTL studies using single-cell RNA-sequencing data have struggled to identify regulatory variants associated with variability [60] since they have focused on studying this phenomenon *in cis*.

## Methods

### Flow cytometry data processing and immune cell gating

Flow cytometry data on TwinsUK participants were downloaded from FlowRepository.org (February 2018) in FCS 3.1 format. Flow cytometry data from the Milieu Intérieur cohort were provided directly by the Milieu Intérieur consortium. A total of 17455 FCS files were processed across both cohorts, with each file representing flow cytometry measurements for a single individual and a specific antibody panel (see S1 Table). The gating schema for each antibody panel (S20–S34 Figs) followed the original study designs for consistency. Prior to cell gating we removed samples with < 1000 recorded events in total. Non-scatter based fluorescence parameter measurements were transformed onto a common scale using a biexponential transform implemented in the R package *flowCore* [62]. To reduce the effects of confounding between technical factors and fluorescence measurements we performed normalization between individuals within an antibody panel (using a warping function estimated from the data) to align feature landmarks for each flow cytometer channel. Function parameter values were set for each target protein, including the number of principal landmarks (*peakNr*), number of spline sections to approximate the expression profile for each protein (*nbreaks*), and the bandwidth of the smooth density estimate (*bwFac*). Subsequently, for each cell type defined by the gating schema, we extracted the fluorescence values across all recorded parameters (protein and scatter-based). For each individual we removed measurements on each cell type where there were fewer than 100 cells. All flow cytometry processing used the *flowCore*, *flowWorkspace*, *flowStats and ggcyto* packages implemented in R [63].

### Protein expression variability calculation

Single cell protein fluorescence measurements for each individual were log_10_ transformed and normalized to cell volume. Cell volume was calculated as the log_10_ of the cubed forward-scatter area. Protein expression variability was calculated across all single cells in each cell type for each individual using the squared coefficient of variation, i.e. variance divided by the squared mean, ${CV}^{2}=\frac{\sigma^{2}}{\mu^{2}}$. The mean-adjusted measure of noise, denoted η_res_, was calculated for each combination of protein, cell type and individual to yield a single trait value. Briefly, a local polynomial regression was used to estimate the mean-CV^2^ relationship across individuals for a given protein expressed in a specific cell type (see S1 Note). The residuals from this fit were standardized, that is they were rescaled to 0 mean and variance of 1, across individuals. Therefore, the final measure of protein expression variability, η_res_, is expressed in terms of the number of relative standard deviations of the residual mean-adjusted CV^2^.

### Genome-wide genotyping and processing

Imputed genome-wide genotyping on TwinsUK participants were provided by the TwinsUK Data Access Committee. Genotypes were imputed using IMPUTE2 [64] as previously described [25], using the 1000 Genomes phase 3 EUR reference panel [65]. Imputed genome-wide genotypes from the Milieu Intérieur cohort were obtained from the European Genome-Phenome archive, accession number EGAD00010001489, approved by the Data Access Committee (DAC). Imputed genotypes, generated by IMPUTE2 from the 1000 Genomes phase 1 EUR reference panel [66], were also downloaded. Binary genotype files in Plink format [38] were used as input for all analyses. Genetic relationship matrices (GRM) were calculated for each cohort of participants using autosomal SNPs. Genetic variants with a cohort minor allele frequency (MAF) < 1% and/or a Hardy-Weinberg Equilibrium ($\chi_{2}^{2}$) p-value ≤1x10^-50^ were excluded from all analyses. For the linear mixed model-based genetic association testing, separate GRMs were pre-computed using genetic variants on each chromosome (A_*chromosome*_), as well as the complementary set of genetic variants, i.e. all genetic variants *not* on the chromosome in question. All GRMs were calculated using *GCTA* [67].

### Variance components analysis and heritability estimation

Variance components analysis of each protein expression mean and variability trait was performed in the TwinsUK cohort. An expected genetic relationship matrix was calculated across all twins, with entries defined by twin zygosity, i.e. 1 for monozygotic twins, 0.5 for dizygotic twins and 0 for unrelated individuals. A second shared environment matrix contained a 1 for twin pairs and 0 for unrelated individuals. These matrices were included as random effects in a model to partition the trait variance into additive genetic (A), common environment (C; indistinguishable from non-additive genetic components) and unique environmental (E) components. Variance decomposition was performed in a structural equation modelling framework, implemented in the R package *umx* [68], which uses a Cholesky decomposition to estimate the model (variance) components as a fraction of the total variance. Variance component standard errors were estimated by a non-parametric bootstrapping procedure using a random sample of 75% of twin pairs. Permutation p-values were computed for each variance component by generating a null distribution of variance component estimates by randomly permuting the twin zygosity labels 100 times for each trait. P-values were then calculated as: $p=1-\left(\frac{\#test>null+1}{\#permutation+1}\right)$.

### Variability-quantitative trait loci genome-wide analysis

Variability-pQTLs were identified genome-wide for each protein expression variability trait using a linear mixed model. Each genetic variant was regressed on trait values measured across individuals, accounting for genetic relatedness between individuals (twins and “unrelated” individuals separately), as well as individual-level covariates. Specifically, a linear mixed model was fit for each trait:

$$y_{i}=\alpha +g\gamma +X\beta +Zu+\epsilon$$

Where *y_i_* is a vector of expression variability trait values (*η_res_*) for trait *i*, *α* is the model intercept, *g* is a vector of SNP genotypes encoded as an additive model (0, 1, 2 copies of the minor allele), *γ* is the fixed effect maximum-likelihood coefficient estimate of the genetic variant on η_res_, *X* is a matrix of fixed-effect covariates, *β* is a vector of maximum-likelihood coefficient estimates for the fixed-effect covariates, *Z* is a genetic covariance matrix calculated from autosomal genetic variants not on the chromosome encoding the protein of interest, *u* is the random-effects coefficient associated with this genetic covariance matrix, and *ϵ* is the residual trait variance. The matrix *X* contains in its columns age (years) and *FCGR2A* rs4657041 genotype (see S3 Note). We tested if there was sufficient evidence to reject the null hypothesis that the SNP effect *γ* = 0, using a t-test.

For *cis*-pQTL testing we extracted all genetic variants within a 1Mb window centered on the transcriptional start site of the gene encoding the target protein, and tested for a SNP-effect using LIMIX [37]. We adjusted for multiple testing first across genetic variants for each *cis* window using a beta-approximation to a permutation null distribution [69], then using a false-discovery control for the total number of traits tested [70]. *Trans*-pQTLs were tested for genome-wide using the same model described above implemented in GCTA [67].

Discrete genetic association signals and lead genetic variants (varSNPs) were assigned at each locus using an LD-based clumping procedure implemented in Plink v1.9. Index variants were selected with a test p-value ≤ 1x10^-4^ for *trans* associations and FDR ≤ 0.05 for *cis*. Additional variants were assigned to clumps within 250kb and r^2^>0.5 of each index variant.

### Mendelian randomization analysis

*Cis*-eQTLs have the potential to drive the *trans*-variability QTLs we identify. For each variability-pQTL we extracted the *cis*-eQTL summary statistics in a 200kb window with a test p-value ≤ 1x10^-5^ for matching cell types (S3 Table). Where overlapping SNPs were present from both data sets we tested the hypothesis of a causal relationship (or shared genetic predictor) between the variability-pQTL and *cis*-eQTL signals. Mendelian Randomization (MR) uses the random assortment of alleles during meiosis as a conditioning factor to determine causal relationships from observational data [71,72]. To assign a meaningful causal relationship between a modifiable exposure (gene expression) and an outcome (eGene expression variability) requires 3 assumptions about the genetic variant (instrumental variable): 1) association between the genetic variant and exposure, 2) uncorrelated with any confounding effects between the exposure and outcome, and 3) conditionally independent of the outcome, given the exposure and confounders. Based on these assumptions, and a linear relationship for all associations, the unbiased causal effect of gene expression on expression variability can be estimated as the ratio of the linear model per-allele effect estimates:

$$\hat{\beta_{causal}}=\frac{\hat{\beta_{outcome}}}{\hat{\beta_{exposure}}}$$

This causal effect can be estimated directly from summary statistics in independent cohorts, known as 2-sample MR [73]. For each eGene and variability-pQTL pair we estimated the causal effect estimate (*β_causal_*) using the MR maximum likelihood approach implemented in the R package *MendelianRandomization* [74]. In analyses where summary statistics were available for multiple SNPs for each trait we combined effect estimates across SNPs using MR-Egger regression [75], implemented in the R package *MendelianRandomization*. In the latter case, we also report Cochrane’s Q-statistic, a measure of genetic instrument heterogeneity as an indication of horizontal pleiotropy [76] (S36 Fig).

### Sensitivity analysis

We determined the sensitivity of both *cis* and *trans* QTL mapping analyses to changes in sample size by down-sampling the number of individuals for a specific trait and repeating the analysis as described above. We randomly selected between 10 and 100% of unrelated individuals from the Milieu Intérieur cohort for two traits for which we had detected both *cis* and *trans* pQTLs: FcεR1A on basophils as a mean trait and HLA-DR on plasmacytoid DCs as a variability trait. Sensitivity was determined as the proportion of QTLs recovered compared to the full sample size. Results are presented in S37 Fig.

## Supporting information

S1 Note Calculation of a mean-adjusted measure of protein expression variability. (DOCX)

S2 Note IgG receptors, genetics and interactions with experimental reagents. (DOCX)

S3 Note Non-genetic influences on protein expression variability. (DOCX)

S4 Note Sample size and power to detect pQTLs. (DOCX)

S1 Table Immune cell flow cytometry definitions. (DOCX)

S2 Table Matching cell types between variability-pQTLs and *cis*-eQTLs. (DOCX)

S3 Table Full results from the Mendelian Randomisation analysis. Listed are the lead VarSNP, MR analysis summary statistics (Beta, SE, P-value), eGene data set, MR heterogeneity statistics and variability-pQTL summary statistics, position and gene information.
(CSV)

S1 Fig TwinsUK Ab Panel 1 variance components analysis. Plotted are proportion of phenotypic variance of components for additive genetic (green), common environment (orange) and unique environment (blue). Each trait is listed on the y-axis for mean (left) and variability (right) traits.
(EPS)

S2 Fig TwinsUK Ab Panel 2 variance components analysis. Plotted are proportion of phenotypic variance of components for additive genetic (green), common environment (orange) and unique environment (blue). Each trait is listed on the y-axis for mean (left) and variability (right) traits.
(EPS)

S3 Fig TwinsUK Ab Panel 3 variance components analysis. Plotted are proportion of phenotypic variance of components for additive genetic (green), common environment (orange) and unique environment (blue). Each trait is listed on the y-axis for mean (left) and variability (right) traits.
(EPS)

S4 Fig TwinsUK Ab Panel 4 variance components analysis. Plotted are proportion of phenotypic variance of components for additive genetic (green), common environment (orange) and unique environment (blue). Each trait is listed on the y-axis for mean (left) and variability (right) traits.
(EPS)

S5 Fig TwinsUK Ab Panel 5 variance components analysis. Plotted are proportion of phenotypic variance of components for additive genetic (green), common environment (orange) and unique environment (blue). Each trait is listed on the y-axis for mean (left) and variability (right) traits.
(EPS)

S6 Fig TwinsUK Ab Panel 6 variance components analysis. Plotted are proportion of phenotypic variance of components for additive genetic (green), common environment (orange) and unique environment (blue). Each trait is listed on the y-axis for mean (left) and variability (right) traits.
(EPS)

S7 Fig TwinsUK Ab Panel 7 variance components analysis. Plotted are proportion of phenotypic variance of components for additive genetic (green), common environment (orange) and unique environment (blue). Each trait is listed on the y-axis for mean (left) and variability (right) traits.
(EPS)

S8 Fig Variability trait changes associated with age and gender in the Milieu Intérieur cohort. (a) Scatter plot of effect sizes from a robust linear regression model of variability (x-axis) and mean (y-axis) with age (years). (b) Traits for which age increases variability (FDR 1%). (c) Traits for which variability decreases with age (FDR 1%). (d) Scatter plot of effect sizes of the influence of gender on expression variability (x-axis) and mean expression (y-axis). (e) Traits (x-axis) for which variability is increased in males relative to females. (f) Variability which are decreased in males relative to females. Purple points in (a) and (b) are associations only with variability, blue points are associations only with mean traits, and orange points are associations with both mean and variability. Points in (b, c, e, f) are regression model effect sizes with 95% confidence intervals.
(EPS)

S9 Fig Variability trait changes associated with age in the TwinsUK cohort. Variability traits that increase (top) and decrease (bottom) with age (FDR 1%). Points are regression model coefficients; error bars are 95% confidence intervals.
(EPS)

S10 Fig Mean expression changes associated with age and gender in the Milieu Intérieur cohort. Mean expression traits that decrease (a) and increase (b) with age. Mean expression traits that are lower (c) and higher (d) in males than females. Points are model regression coefficients; error bars are 95% confidence intervals.
(EPS)

S11 Fig Additive genetic component estimates for heritable expression variability traits from variance components analysis. Error bars represent bootstrapped standard errors from 100 permutations. Orange points are those with h^2^ +/- SE that fall within the interval [0, 1] (defined by the light grey box).
(EPS)

S12 Fig Histogram of permutation p-values for additive genetic variance components. Distribution of empirical p-values of additive genetic variance components for expression variability traits.
(EPS)

S13 Fig Variance components analysis calculated values plotted by protein. Variance components estimates grouped by protein for mean (blue) and variability (orange) traits across cell types. Y-axis shows the % phenotypic variance, X-axis shows the variance components (A-additive genetic, C-common environment, E-unique environment).
(EPS)

S14 Fig TwinsUK variability-pQTL Manhattan plot. Linear mixed model -log_10_ association p-values (y-axis) between expression variability and genome-wide genetic variants (x-axis). The purple horizontal line represents genome-wide significance threshold (5x10^-8^), and the orange line represents the Bonferroni corrected threshold (8.47x10^-10^).
(EPS)

S15 Fig TwinsUK mean-pQTL Manhattan plot. Linear mixed model -log_10_ association p-values (y-axis) between expression variability and genome-wide genetic variants (x-axis). The purple horizontal line represents genome-wide significance threshold (5x10^-8^), and the orange line represents the Bonferroni corrected threshold (8.47x10^-10^).
(EPS)

S16 Fig Milieu Intérieur variability-pQTL Manhattan plot. Linear mixed model -log_10_ association p-values (y-axis) between expression variability and genome-wide genetic variants (x-axis). The purple horizontal line represents genome-wide significance threshold (5x10^-8^), and the orange line represents the Bonferroni corrected threshold (8.47x10^-10^).
(EPS)

S17 Fig Milieu Intérieur mean-pQTL Manhattan plot. Linear mixed model -log_10_ association p-values (y-axis) between mean expression levels and genome-wide genetic variants (x-axis). The purple horizontal line represents genome-wide significance threshold (5x10^-8^), and the orange line represents the Bonferroni corrected threshold (8.47x10^-10^).
(EPS)

S18 Fig Overlap of genetically regulated mean and variability traits between Milieu Intérieur and TwinsUK cohorts. Venn diagrams showing the overlap of mean (a) and expression variability traits (b) between the Milieu Intérieur (red) and TwinsUK (blue).
(EPS)

S19 Fig Mendelian Randomization results top causal relationships. Shown are the MR regression estimate (β), error bars denote the 95% CI for relationships at FDR 10%. Y-axis labels show the vProtein and eGene. Points are coloured by the cell type in which the eGene and variability QTL are both present.
(EPS)

S20 Fig Flow cytometry gating schemes for each cell type in the TwinsUK cohort—Ab Panel 1. (EPS)

S21 Fig Flow cytometry gating schemes for each cell type in the TwinsUK cohort—Ab Panel 2. (EPS)

S22 Fig Flow cytometry gating schemes for each cell type in the TwinsUK cohort—Ab Panel 3. (EPS)

S23 Fig Flow cytometry gating schemes for each cell type in the TwinsUK cohort—Ab Panel 4. (EPS)

S24 Fig Flow cytometry gating schemes for each cell type in the TwinsUK cohort—Ab Panel 5. (EPS)

S25 Fig Flow cytometry gating schemes for each cell type in the TwinsUK cohort—Ab Panel 6. (EPS)

S26 Fig Flow cytometry gating schemes for each cell type in the TwinsUK cohort—Ab Panel 7. (EPS)

S27 Fig Flow cytometry gating schemes for each cell type in the Milieu Intérieur cohort—Ab Panel 1. (EPS)

S28 Fig Flow cytometry gating schemes for each cell type in the Milieu Intérieur cohort—Ab Panel 2. (EPS)

S29 Fig Flow cytometry gating schemes for each cell type in the Milieu Intérieur cohort—Ab Panel 3. (EPS)

S30 Fig Flow cytometry gating schemes for each cell type in the Milieu Intérieur cohort—Ab Panel 4. (EPS)

S31 Fig Flow cytometry gating schemes for each cell type in the Milieu Intérieur cohort—Ab Panel 5. (EPS)

S32 Fig Flow cytometry gating schemes for each cell type in the Milieu Intérieur cohort—Ab Panel 7. (EPS)

S33 Fig Flow cytometry gating schemes for each cell type in the Milieu Intérieur cohort—Ab Panel 8. (EPS)

S34 Fig Flow cytometry gating schemes for each cell type in the Milieu Intérieur cohort—Ab Panel 9. (EPS)

S35 Fig Manhattan plots of negative control trait CD3 expression variability in neutrophils before and after adjustment for *FCGR2A* genotype. Linear mixed model -log_10_ association p-values (y-axis) between CD3 expression variability in granulocytes and genome-wide genetic variants (x-axis) without (top) and with (bottom) adjustment for *FCGR2A* genotype at rs4657041. The purple horizontal line represents genome-wide significance threshold (5x10^-8^), and the orange line represents the Bonferroni corrected threshold (8.47x10^-10^).
(EPS)

S36 Fig Mendelian Randomization heterogeneity from MR-Egger regression analyses for top 5% of causal relationships. Shown are the Cochrane’s Q-values from the MR-Egger regression across SNPs (x-axis) for each pair of varSNP and eGene (y-axis). Points are coloured by the broad matching cell type between the *trans* variability-pQTL and *cis*-eQTL. Point size is proportional to the -log_10_ p-value from a χ^2^ test.
(EPS)

S37 Fig Sensitivity analysis of *cis* and *trans* pQTL mapping for mean and variability traits. Shown are the proportion of QTLs detected (y-axis) as a function of the sample size (x-axis) for both mean (salmon) and variability (turquoise) traits. (a) Cis-QTL mapping and (b) trans-QTL mapping were performed separately. Numbers denote the total number of QTLs detected with the largest sample size.
(EPS)
