# Supplementary material for: Quantitative genetic analysis deciphers the impact of cis and trans regulation on cell-to-cell variability in protein expression levels
Source: PLoS Genet. 2020 Mar 13;16(3):e1008686. doi: 10.1371/journal.pgen.1008686 (PMC7094872; doi:10.1371/journal.pgen.1008686)
Supplement: S1 Note — (DOCX) [file pgen.1008686.s001.docx]

**Supplementary Note 1: Calculation of a mean-adjusted measure of protein expression variability**

In studies of mRNA or protein expression variability it is critical to account for the relationship between average expression levels and variability. The exact nature of the adjustments required to control for this relationship largely depends on the technology used for quantification. In the context of flow cytometry data, although noise models have been described[1], these rely on assumptions about laser geometry and the plane in which cells are measured as they flow through the laser path, antibody binding kinetics and flow cytometer voltages. Given that such information is typically unavailable (including for the datasets profiled in this study), we used a data-driven approach to model the relationship between mean expression and variability. Our model assumes that technical and experimental sources of variability are orthogonal to the biological variables of interest, i.e. age, genetics, etc.

We use the squared coefficient of variation (CV^2^) as our measure of variability as it is scale invariant. To account for the well-known observation that the CV^2^ decreases as a function of the mean (see Ziv Bar-Even *et al*. for a discussion of this relationship[2]), for each protein in each cell type we fit a local polynomial regression (loess) across all individuals with non-missing data. We then use this fit to calculate the residual variation in the CV^2^ for each individual for a given protein and cell type. We use the same bandwidth (span) in the curve fitting process for each protein. To standardize the residuals, we performed a Z-score standardisation across individuals for each protein and cell type variability trait to derive our final measure of protein expression variability, η_res_. This provides an interpretation for model coefficients in units of standard deviations. These steps are formalised below:

${CV}^{2}(\mu)= \alpha+s\left( \mu\right)+\epsilon$ (1)

Where s(μ) is a locally-fitted weighted least squares function, α is the model intercept, and ε is the residual variance. Therefore, the residual CV^2^ is calculated from this model fit for individual *i*:

${rCV}_{i}^{2}={CV}_{i}^{2}-E\left[ {CV}^{2} \right]$ (2)

Finally, we standardise these residual values across individuals:

$\eta_{res}=\frac{{rCV}_{i}^{2}-\bar{{rCV}^{2}}}{\sigma^{2}}$ (3)

Where $\bar{{rCV}^{2}}$ is the mean residual CV^2^ across individuals for that protein and cell type, and $\sigma^{2}$ is the variance of residual CV^2^ values across the same individuals for the relevant protein and cell type. Our mean-adjusted measure of variability is not correlated with mean expression level (Figure 1b).

References

1. Ubezio P, Andreoni A. Linearity and noise sources in flow cytometry. Cytometry. 1985;6: 109–115. doi:10.1002/cyto.990060205

2. Bar-Even A, Paulsson J, Maheshri N, Carmi M, O’Shea E, Pilpel Y, et al. Noise in protein expression scales with natural protein abundance. Nat Genet. 2006;38: 636–643. doi:10.1038/ng1807
