# Supplementary material for: Quantitative genetic analysis deciphers the impact of cis and trans regulation on cell-to-cell variability in protein expression levels
Source: PLoS Genet. 2020 Mar 13;16(3):e1008686. doi: 10.1371/journal.pgen.1008686 (PMC7094872; doi:10.1371/journal.pgen.1008686)
Supplement: S2 Note — (DOCX) [file pgen.1008686.s002.docx]

**Supplementary Note 2: IgG receptors, genetics and interactions with experimental reagents**

To establish that our genetic association study of protein variability did not detect any confounding between technical or unwanted experimental sources of variability and genetic variation, we included a negative control trait in our analyses. Specifically, we selected a protein that is known to not be expressed in a particular cell type: CD3 in neutrophils. CD3 expression is restricted to particular lymphoid cells, i.e. T cells, NK cells and NKT cells. Therefore, the detection of genetic variants that are associated with CD3 in these myeloid cells suggests potential confounding between biological and technical factors. We performed a GWAS of CD3 variability in neutrophils using a LMM:

$\eta_{res}=\alpha+g\gamma+X\beta+Zu+\epsilon$ (4)

Where α is the model intercept, *g* is a vector of SNP genotypes encoded as an additive model (0, 1, 2 copies of the minor allele), γ is the corresponding coefficient, *X* is a matrix of fixed-effect covariates, β is the corresponding coefficient, *Z* is a genetic covariance matrix calculated from autosomal genetic variants not on the chromosome encoding the protein of interest, u is the random-effects coefficient associated with this genetic covariance matrix, and ε is the residual trait variance. The vector *X* contains the age (in years) of each sample. This model is implemented in GCTA[1] and LIMIX[2], and the null hypothesis test is that the SNP effect γ = 0, using a t-test.

We found that a single locus achieved genome-wide statistical significance (p-value ≤ 5x10^-8^) in this analysis of our negative control trait (Supplementary Figure 35). The lead SNP for this association signal was rs4657041 which maps to the 3rd intron of *FCGR2A*; this gene encodes the class IIa Fcγ receptor. This important class of immune receptors are expressed primarily on myeloid phagocytic cells, such as neutrophils, and bind immunoglobulin G to elicit immune responses, crucial for fighting bacterial and viral pathogens. Importantly, fluorescent molecule-conjugated IgG antibodies (Ab) are used in flow cytometry to target molecules of interest for quantification. Commonly, a blocking antibody is added to prevent non-specific binding of IgG Abs to these Fcγ receptors. This was the case for all samples in both the TwinsUK and Milieu Intérieur cohorts. However, the minor allele of this SNP is associated with elevated *FCGR2* plasma levels[3]. Thus, this association represents a likely interaction between a polymorphism that segregates in nearly 50% of European individuals (study MAF 49.1%, T>C), and an experimental reagent. Delineating the exact mechanism by which this genetic variant leads to altered binding dynamics is beyond the scope of this manuscript. However, it does demonstrate the importance of including negative control traits in studies that make use of antibody-binding based assays in cells derived from human participants.

Subsequently, we adapted our LMM (4) to include individual-level rs4657041 genotypes as an additional fixed effect in the matrix *X* to analytically account for this confounding interaction between genetics and experimental reagent. We found that this adequately removed the observed association between the *FCGR2A* locus and CD3 variability in neutrophils (Supplementary Figure 35).

References

1. Yang J, Lee SH, Goddard ME, Visscher PM. GCTA: A Tool for Genome-wide Complex Trait Analysis. Am J Hum Genet. 2011;88: 76–82. doi:10.1016/j.ajhg.2010.11.011

2. Casale FP, Rakitsch B, Lippert C, Stegle O. Efficient set tests for the genetic analysis of correlated traits. Nat Methods. 2015;12: 755–758. doi:10.1038/nmeth.3439

3. Sun BB, Maranville JC, Peters JE, Stacey D, Staley JR, Blackshaw J, et al. Genomic atlas of the human plasma proteome. Nature. 2018;558: 73–79. doi:10.1038/s41586-018-0175-2
