# Supplementary material for: Quantitative genetic analysis deciphers the impact of cis and trans regulation on cell-to-cell variability in protein expression levels
Source: PLoS Genet. 2020 Mar 13;16(3):e1008686. doi: 10.1371/journal.pgen.1008686 (PMC7094872; doi:10.1371/journal.pgen.1008686)
Supplement: S3 Note — (DOCX) [file pgen.1008686.s003.docx]

**Supplementary Note 3: Non-genetic influences on protein expression variability** Shared non-genetic factors between twins, including lifestyle and environment, modulate cell-to-cell gene expression variability (Figure 2a). To explore this, we considered the Milieu Intérieur cohort and used a multivariate linear model to investigate the impact of age and gender on protein expression variability, while controlling for *FCGR2A* genotype and CMV serostatus (Methods). Our analysis identified 90 expression variability traits at a 1% FDR that changed with age (Supplementary Figure 8a).

Amongst the proteins that increase expression variability with age was CD3, which is critical for T cell activation and signalling through the TCR. This highly variable CD3 expression is present in naive and memory subsets of CD8+ T cells (Supplementary Figure 8b). Providing independent support for this observation, increased CD3 variability was also identified in the smaller TwinsUK cohort (Supplementary Figure 9). These findings are consistent with a report illustrating more heterogeneous T cell activation with age in mice, using an anti-CD3 antibody as stimulation[1]. CD3 expression becomes more variable with age in these mice, suggesting highly variable CD3 expression on human T cells may lead to less efficient activation with advancing age.

In addition to greater expression heterogeneity, we observed a reduction in variability for many proteins, including CD8 on CD8+ T cell subsets (Supplementary Figure 8b), and functional receptors on innate immune cells (CD32 - eosinophils, CD14 - monocytes). The frequency of CD14+ monocytes also declines with age, with a concomitant increase in CD16+ non-classical monocytes[2]. Additionally, a small number of proteins show a consistent reduction in variability across multiple lineages, suggesting the potential for age-related effects that act in a common precursor, or by a shared molecular mechanism. For instance, CD8 is down-regulated with age in multiple T cell subsets, including specialized sub-types (CD8+ Tc1), memory cells (TEM, TCM) and rare CD4+CD8+ T cells.

In addition to the effects of age on protein expression variability, we identified 28 traits that differed between male and female participants from the Milieu Intérieur cohort (Supplementary Figure 8c-e). These changes included greater CD45RA expression variability on CD4+ effector memory T cells in females, as well as less variable CD45RA on naive Tregs.

References

1. Martinez-Jimenez CP, Eling N, Chen H-C, Vallejos CA, Kolodziejczyk AA, Connor F, et al. Aging increases cell-to-cell transcriptional variability upon immune stimulation. Science. 2017;355: 1433–1436. doi:10.1126/science.aah4115

2. Patin E, Bergstedt J, Rouilly V, Libri V, Urrutia A, Alanio C, et al. Natural variation in the parameters of innate immune cells is preferentially driven by genetic factors. Nat Immunol. 2018;19: 302–314. doi:10.1038/s41590-018-0049-7
