# Supplementary material for: Quantitative genetic analysis deciphers the impact of cis and trans regulation on cell-to-cell variability in protein expression levels
Source: PLoS Genet. 2020 Mar 13;16(3):e1008686. doi: 10.1371/journal.pgen.1008686 (PMC7094872; doi:10.1371/journal.pgen.1008686)
Supplement: S4 Note — (DOCX) [file pgen.1008686.s004.docx]

**Supplementary Note 4: Sample size and power to detect pQTLs**

Sarkar *et al* recently reported that a higher signal-to-noise ratio for gene expression variability relative to mean gene expression reduces power to detect *cis* variability-eQTLs [1]. To examine whether this phenomenon was replicated in the context of our flow cytometry data, we performed a sensitivity analysis (Supplementary Figure 37). Specifically, we considered one mean trait and one (non-overlapping) variability trait, for which both *cis* and *trans*-pQTLs were detected in our analysis. For the mean trait, we observed that 75% of *cis*-pQTLs and 50% of trans-pQTLs were identified when sampling 50% of individuals. By contrast, for the variability trait, we noted that the power to detect pQTLs acting either in trans or in cis declined sharply but consistently for both regulatory mechanisms. Although limited by the very small number of traits for which both cis and trans variants were identified, this analysis suggests that our relative ability to detect variability pQTLs is more strongly dependent upon sample size than our ability to detect mean pQTLs, consistent with the results presented by Sarkar *et al*. [1].

References

1. Sarkar AK, Tung P-Y, Blischak JD, Burnett JE, Li YI, Stephens M, et al. Discovery and characterization of variance QTLs in human induced pluripotent stem cells. Cotsapas C, editor. PLOS Genet. 2019;15: e1008045. doi:10.1371/journal.pgen.1008045
