# Supplementary material for: Quantitative genetic analysis deciphers the impact of cis and trans regulation on cell-to-cell variability in protein expression levels
Source: PLoS Genet. 2020 Mar 13;16(3):e1008686. doi: 10.1371/journal.pgen.1008686 (PMC7094872; doi:10.1371/journal.pgen.1008686)
Supplement: S1 Table — (DOCX) [file pgen.1008686.s005.docx]

| Cohort | Ab panel | Cell type | FACS phenotype |
| --- | --- | --- | --- |
| TwinsUK | P1 | CD8+ SCM T cell | CD3+CD8+CCR7+CD45RA^hi^CD95^hi^ |
| TwinsUK | P1 | CD8+ CM T cell | CD3+CD8+CCR7+CD45RA-CD28- |
| TwinsUK | P1 | CD8+ TTM T cell | CD3+CD8+CCR7-CD45RA+CD95- |
| TwinsUK | P1 | CD8+ EM T cell | CD3+CD8+CCR7-CD95+ |
| TwinsUK | P1 | CD8+ Naïve T cell | CD3+CD8+CCR7+CD127+ |
| TwinsUK | P1 | CD4+ SCM T cell | CD3+CD4+CCR7+CD45RA^hi^CD95^hi^ |
| TwinsUK | P1 | CD4+ CM T cell | CD3+CD4+CCR7+CD45RA-CD28- |
| TwinsUK | P1 | CD4+ TTM T cell | CD3+CD4+CCR7-CD45RA+CD95- |
| TwinsUK | P1 | CD4+ EM T cell | CD3+CD4+CCR7-CD95+ |
| TwinsUK | P1 | CD4+ Naïve T cell | CD3+CD4+CCR7+CD127+ |
| TwinsUK | P1 | CD4+CD8+ DP T cell | CD3+CD4+CD8+ |
| TwinsUK | P1 | CD4-CD8- DN T cell | CD3+CD4-CD8- |
| TwinsUK | P1 | Recent thymic emigrants | CD3+CD31^hi^D45RA^hi^ |
| TwinsUK | P2 | CD4+CD8+ DP T cell | CD3+CD4+CD8+ |
| TwinsUK | P2 | CD4+ Exhausted T cell | CD3+CD4+CD45RO+PD-1+ |
| TwinsUK | P2 | CD4+ Treg | CD3+CD4+CD25+ |
| TwinsUK | P2 | CD4-CD8- DN T cell | CD3+CD4-CD8- |
| TwinsUK | P2 | CD8+ Treg | CD3+CD8+CD25+ |
| TwinsUK | P3 | CD4+ CD8+ DP T cell | CD3+CD4+CD8+ |
| TwinsUK | P3 | CD4+ T cell | CD3+CD4+ |
| TwinsUK | P3 | CD8+ T cell | CD3+CD8+ |
| TwinsUK | P3 | CD4-CD8- DN T cell | CD3+CD4-CD8- |
| TwinsUK | P4 | CD3- NK cell | CD3+CD1-multi+ |
| TwinsUK | P5 | HSC | CD3-CD34^hi^ |
| TwinsUK | P5 | Vγ9+Vδ2^hi^ γδ T cell | CD3+Vg9+Vd2^hi^ |
| TwinsUK | P5 | Vγ9+ Vδ2^lo^ γδ T cell | CD3+Vg9+Vd2^lo^ |
| TwinsUK | P5 | Vδ1+ γδ T cell | CD3+VD1-Vd2- |
| TwinsUK | P5 | Vγ9+ Vδ1- γδ T cell | CD3+Vg9+Vd1- |
| TwinsUK | P6 | IgM Memory B cell | CD19+CD10-CD95+IgA-IgG-IgM+ |
| TwinsUK | P6 | Naive mature B cell | CD19+CD10-CD95- |
| TwinsUK | P6 | IgG Memory B cell | CD19+CD10-CD95+IgA-IgG+ |
| TwinsUK | P6 | IgA Memory B cell | CD19+CD10-CD95+IgA+IgG- |
| TwinsUK | P6 | IgE Memory B cell | CD19+CD10-CD95+IgM-IgD- |
| TwinsUK | P6 | Immature B cell | CD19+CD10+ |
| TwinsUK | P7 | Plasmacytoid DC | HLA-DR+CD14-CD123+CD11c- |
| TwinsUK | P7 | CD123+CD11c+ Myeloid DC | HLA-DR+CD14-CD123+CD11c+ |
| TwinsUK | P7 | CD4 cDC1 | HLA-DR+CD14-CD123-CD11c+CD141- |
| TwinsUK | P7 | CD8 cDC1 | HLA-DR+CD14-CD123-CD11c+CD141+ |
| TwinsUK | P7 | CD14+ monocyte | HLA-DR+CD14^hi^ |
| TwinsUK | P7 | CD16+ monocyte | HLA-DR+CD14^lo^CD16+ |
| Milieu Intérieur | P1 | CD4+ CD8+ DP T cell | CD3+CD4+CD8+ |
| Milieu Intérieur | P1 | CD4- CD8- DN T cell | CD3+CD4-CD8- |
| Milieu Intérieur | P1 | CD4+ Naïve T cell | CD3+CD4+CD27+CD45RA+ |
| Milieu Intérieur | P1 | CD8+ Naïve T cell | CD3+CD8+CD27+CD45RA+ |
| Milieu Intérieur | P1 | CD4+ Central Memory T cell (TCM) | CD3+CD4+CD27+CD45RA- |
| Milieu Intérieur | P1 | CD8+ Central Memory T cell (TCM) | CD3+CD8+CD27+CD45RA- |
| Milieu Intérieur | P1 | CD4+ Effector Memory T cell (EM) | CD3+CD4+CD27-CD45RA- |
| Milieu Intérieur | P1 | CD8+ Effector Memory T cell (EM) | CD3+CD8+CD27-CD45RA- |
| Milieu Intérieur | P1 | CD4+ Effector Memory RA T cell (EMRA) | CD3+CD4+CD27-CD45RA+ |
| Milieu Intérieur | P1 | CD8+ Effector Memory RA T cell (EMRA) | CD3+CD8+CD27-CD45RA+ |
| Milieu Intérieur | P2 | CD4+ CD8+ DP T cell | CD3+CD4+CD8+ |
| Milieu Intérieur | P2 | CD4- CD8- DN T cell | CD3+CD4-CD8- |
| Milieu Intérieur | P2 | CD4+ T cells | CD3+CD4+ |
| Milieu Intérieur | P2 | CD8+ T cells | CD3+CD8+ |
| Milieu Intérieur | P2 | CD4+ Regulatory T cells | CD3+CD4+CD127^lo^CD25+ |
| Milieu Intérieur | P2 | CD8+ Regulatory T cells | CD3+CD8+CD127^lo^CD25+ |
| Milieu Intérieur | P2 | Naive CD4+ Tregs | CD3+CD4+CD127^lo^CD25+HLA-DR-CD45RA- |
| Milieu Intérieur | P2 | Naive CD8+ Tregs | CD3+CD8+CD127^lo^CD25+HLA-DR-CD45RA- |
| Milieu Intérieur | P2 | Activated CD4+ Tregs | CD3+CD4+CD127^lo^CD25+HLA-DR+CD45RA- |
| Milieu Intérieur | P2 | Activated CD8+ Tregs | CD3+CD8+CD127^lo^CD25+HLA-DR+CD45RA- |
| Milieu Intérieur | P2 | Memory CD4+ Tregs | CD3+CD4+CD127^lo^CD25+HLA-DR-CD45RA+ |
| Milieu Intérieur | P2 | Memory CD8+ Tregs | CD3+CD8+CD127^lo^CD25+HLA-DR-CD45RA+ |
| Milieu Intérieur | P3 | CD4+ CD8+ DP T cell | CD3+CD4+CD8+ |
| Milieu Intérieur | P3 | CD4- CD8- DN T cell | CD3+CD4-CD8- |
| Milieu Intérieur | P3 | CD4+ T cell | CD3+CD4+CD8- |
| Milieu Intérieur | P3 | CD8+ T cell | CD3+CD4-CD8+ |
| Milieu Intérieur | P3 | γδ T cell | CD3+TCRγδ+ |
| Milieu Intérieur | P3 | NKT cell | CD3+CD161+TCRV24+ |
| Milieu Intérieur | P3 | MAIT cell | CD3+CD161+TCRV7.2+ |
| Milieu Intérieur | P4 | CD16+CD56^bright^ NK cell | CD3-CD14-NKP46+CD56++ |
| Milieu Intérieur | P4 | CD16+CD56^dim^ NK cell | CD3-CD14-NKP46+CD16+CD56+ |
| Milieu Intérieur | P5 | B cells | CD16-CD19+ |
| Milieu Intérieur | P5 | CD4+CD8+ DP T cells | CD45+CD3+CD4+CD8+ |
| Milieu Intérieur | P5 | CD4+CD8+ DN T cells | CD45+CD3+CD4-CD8- |
| Milieu Intérieur | P5 | CD4+ T cells | CD45+CD3+CD4+CD8- |
| Milieu Intérieur | P5 | CD8+ T cells | CD45+CD3+CD4-CD8+ |
| Milieu Intérieur | P5 | Neutrophils | CD45+CD3-CD16+ |
| Milieu Intérieur | P5 | Classical monocytes | CD45+CD3-CD14++CD16- |
| Milieu Intérieur | P5 | Non-classical monocytes | CD45+CD3-CD14+CD16+ |
| Milieu Intérieur | P5 | CD16+CD56^bright^ NK cell | CD45+CD3-CD56++CD16- |
| Milieu Intérieur | P5 | CD16+CD56^dim^ NK cell | CD45+CD3-CD56+CD16+ |
| Milieu Intérieur | P7 | Neutrophils | CD16+CD125- |
| Milieu Intérieur | P7 | Eosinophils | CD16-CD32+CD125+ |
| Milieu Intérieur | P7 | Basophils | CD16-FcERIA+CD125- |
| Milieu Intérieur | P8 | Monocytes | CD14+HLA-DR+ |
| Milieu Intérieur | P8 | Plasmacytoid DCs | CD14-HLA-DR+BDCA4+BDCA2+ |
| Milieu Intérieur | P8 | Conventional DC 1 | CD14-HLA-DR+BDCA2-BDCA4-BDCA1+ |
| Milieu Intérieur | P8 | Conventional DC 3 | CD14-HLA-DR+BDCA2-BDCA4-BDCA3+ |
| Milieu Intérieur | P9 | γδ T cell | TCRγδ+ |
| Milieu Intérieur | P9 | CD4+CD8+ DP T cells | TCRγδ-CD4+CD8+ |
| Milieu Intérieur | P9 | CD4+CD8+ DN T cells | TCRγδ-CD4-CD8- |
| Milieu Intérieur | P9 | CD4+ T cells | TCRγδ-CD4+CD8- |
| Milieu Intérieur | P9 | CD8+ T cells | TCRγδ-CD4-CD8+ |
| Milieu Intérieur | P9 | CD8+ CCR6+ T cells | TCRγδ-CD4-CD8+CCR6+ |
| Milieu Intérieur | P9 | CD8+ CCR6- T cells | TCRγδ-CD4-CD8+CCR6- |
| Milieu Intérieur | P9 | CD4+ Th17 cells | TCRγδ-CD4+CD8-CCR6+ |
| Milieu Intérieur | P9 | CD4+ Th2 cells | TCRγδ-CD4+CD8-CCR6-CRTh2+CXCR5- |
| Milieu Intérieur | P9 | CD4+ Tfh cells | TCRγδ-CD4+CD8-CCR6-CRTh2-CXCR5+ |
