# Supplementary material for: Quantitative genetic analysis deciphers the impact of cis and trans regulation on cell-to-cell variability in protein expression levels
Source: PLoS Genet. 2020 Mar 13;16(3):e1008686. doi: 10.1371/journal.pgen.1008686 (PMC7094872; doi:10.1371/journal.pgen.1008686)
Supplement: S2 Table — (DOCX) [file pgen.1008686.s006.docx]

| Variability-pQTL cell type | *Cis*-eQTL cell type | Study |
| --- | --- | --- |
| IgA+ Memory B cells | Naive B cell  CD19+ B cell | Schmiedel *et al*  Ishigaki *et al* |
| CD4+ T cells | CD4+ T cells  CD4+ naive T cells, Stimulated CD4+ naive T cells, CD4+ Tfh, CD4+ Th1, CD4+ Th2, CD4+ Th17, CD4+ Memory Treg | Ishigaki *et al*  Schmiedel *et al* |
| CD8+ T cells | CD8+ T cells  CD8+ Naive T cells, Stimulated CD8+ naive T cells | Ishigaki *et al*  Schmiedel *et al* |
| Monocytes | CD14+ Monocytes | Ishigaki *et al*, Schmiedel *et al*, Chen *et al* |
| Granulocytes | PBMC  Neutrophils | Ishigaki *et al*  Chen *et al* |
| NK cells | CD56+ NK cells  NK cells | Ishigaki *et al*  Schmiedel *et al* |
